# How *β*‑Ionone Ring Dynamics and Conformational Heterogeneity Control Carotenoid Photophysics

**DOI:** 10.1021/acs.jpclett.6c02098

**Published:** 2026-09-17

**Authors:** V. K. Jaiswal, L. Uboldi, A. Bonvicini, A. Nenov, M. Garavelli, L. Muccioli, D. Polli, H. Hashimoto, Chiasa Uragami, G. Cerullo, F. Segatta

**Affiliations:** ∇ Dipartimento di Chimica Industriale “Toso Montanari”, Università di Bologna - Alma Mater Studiorum, Via Piero Gobetti 85, 40129 Bologna, Italy; ‡ Dipartimento di Fisica, 18981 Politecnico di Milano, Piazza Leonardo da Vinci 32, I-20133, Milano, Italy; § Department of Applied Chemistry for Environment, Graduate School of Science and Technology, 12907 Kwansei Gakuin University, 1 Gakuen-Uegahara, Sanda 669-1330, Japan; ∥ Theoretical Chemistry Laboratory, Unit of Theoretical and Structural Physical Chemistry, Namur Institute of Structured Matter, 54501 University of Namur, B-5000 Namur, Belgium

## Abstract

Carotenoid ultrafast photophysics has been studied for decades, but fully resolving their excited-state relaxation mechanisms remains a central, driving pursuit. Here, we investigate mini-*β*-carotene (m5) as a tractable model system, coupling high time-resolution transient absorption spectroscopy with ab-initio high-level multireference quantum chemistry calculations. We reveal the key and often overlooked role of the *β*-ionone ring-backbone torsional coordinate, which acts as a dynamic “conjugation length control knob” during excited-state relaxation. The torsion of these rings produces ground-state conformational heterogeneity that shapes excited-state spectroscopic signatures and governs the non-radiative relaxation pathway. Explicitly accounting for this coordinate allows us to decipher the ultrafast dynamics and provide a unified interpretation of the experimental transient absorption spectral features. We introduce a general framework based on ring-backbone coupling that bridges simple polyenes and complex carotenoids across varying chain lengths.

## Graphical abstract

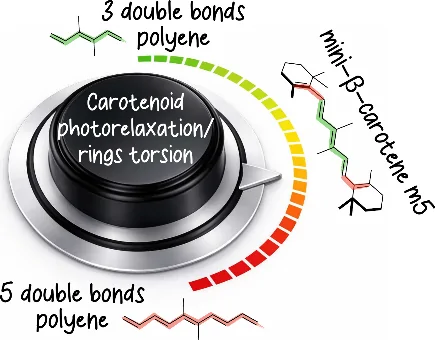

Carotenoids (Cars; singular: Car) are widespread, biologically essential pigments that govern crucial photophysical processes in nature. They act as light-harvesting accessories in photosynthetic apparatuses, photoprotective agents safeguarding systems against singlet oxygen, and regulatory dissipators of excess energy.^1–10^ Their rigid, elongated structures provide structural stabilization to light-harvesting complexes.^11–13^ Recently they have emerged as platforms for studying singlet fission^14–16^ and bleeding-edge organic electronics with on-demand degradation.^17^

Despite decades of study, the exact electronic structure of excited Cars remains a subject of intense debate. Their photophysics is dominated by two lowest singlet excited-states: the optically bright 1^1^ *B*_*u*_ state and the lower-lying, dipole-forbidden 2^1^ *A*_*g*_ dark state, with 1^1^ *A*_*g*_ being the electronic ground-state. Photoexcitation into the 1^1^ *B*_*u*_ state triggers an ultrafast internal conversion (IC) to the 2^1^ *A*_*g*_ state on a 100 fs time scale. Directly mapping the vertical energy of the forbidden 2^1^ *A*_*g*_ state and its chain-length dependence remains exceptionally challenging. For Cars with *N* ≥ 9 (*N* being the number of conjugated double bonds in the polyenic moiety), additional transient signals have prompted proposals of intermediate dark states (S*, S_*X*_) bridging the 1^1^ *B*_*u*_ → 2^1^ *A*_*g*_ IC,^18–23^ though their definitive identification is complicated by possible contributions from ground-state or 2^1^ *A*_*g*_ conformers populated during relaxation.^24,25^

To interpret this behavior, numerous models have historically mapped Car photophysics onto simpler, static polyene chains of equivalent conjugation length *N*.^26^ While computationally efficient, these models typically neglect the dynamic role of the non-planar nuclear framework, particularly the torsion of the lateral *β*-ionone rings relative to the central conjugated backbone. Although ground-state studies have recognized that this ring-backbone twist reduces the effective conjugation length,^27–36^ its dynamic influence on the relative energetics of the excited-states and their explicit relaxation pathways has received significantly less attention.^29,37–39^

Here, we investigate mini-5-*β*-carotene (m5), a system with five conjugated double bonds that is amenable to both high-level ab initio quantum chemistry and state-of-the-art ultrafast spectroscopy. By directly pairing these experiments with simulations at the multi-reference CASSCF/CASPT2 level of theory and explicitly accounting for the ring-backbone torsional coordinates, we unravel the underlying relaxation mechanism. We show that the rotation of the *β*-ionone rings relative to the backbone functions as a dynamic “conjugation length control knob” during the excited-state lifetime, continuously tuning the properties of the Car between shorter and longer polyenic regimes. Furthermore, we demonstrate how ground-state conformational heterogeneity across these torsional degrees of freedom actively dictates the multi-exponential relaxation paths and introduces specific signatures in the transient spectra. Ultimately, this work offers a unified framework that reconciles the photophysics of polyenes and Cars across varying chain lengths.

## m5 Transient Absorption Spectrum

We performed ultrafast transient absorption (TA) spectroscopy on m5 in cyclohexane, utilizing a probe photon energy window of 1.8–3.2 eV and a pump-probe time delay of up to 1 ps (Figure 1a). Following photoexcitation by a sub-30 fs pulse centered at 4.2 eV, the differential absorption (Δ*A*) spectrum exhibits two intense bands: a negative feature at the high-energy edge of the probe window (centered at ∼3.2 eV) and a positive feature at lower energies (spanning 1.8 to 2.2 eV). These signals rise immediately after the photoexcitation pulse and are attributed to the photoexcited bright 1^1^ *B*_*u*_ state, specifically, to its stimulated emission (SE) and first photoinduced absorption (PA) feature (denoted as PA1), respectively. As established by global analysis of the TA spectra (Section S1 of SI), both features decay synchronously with a time constant of 63 ± 5 fs, tracking the ultrafast IC from the bright 1^1^ *B*_*u*_ state to the dark 2^1^ *A*_*g*_ state.

**Figure 1:**
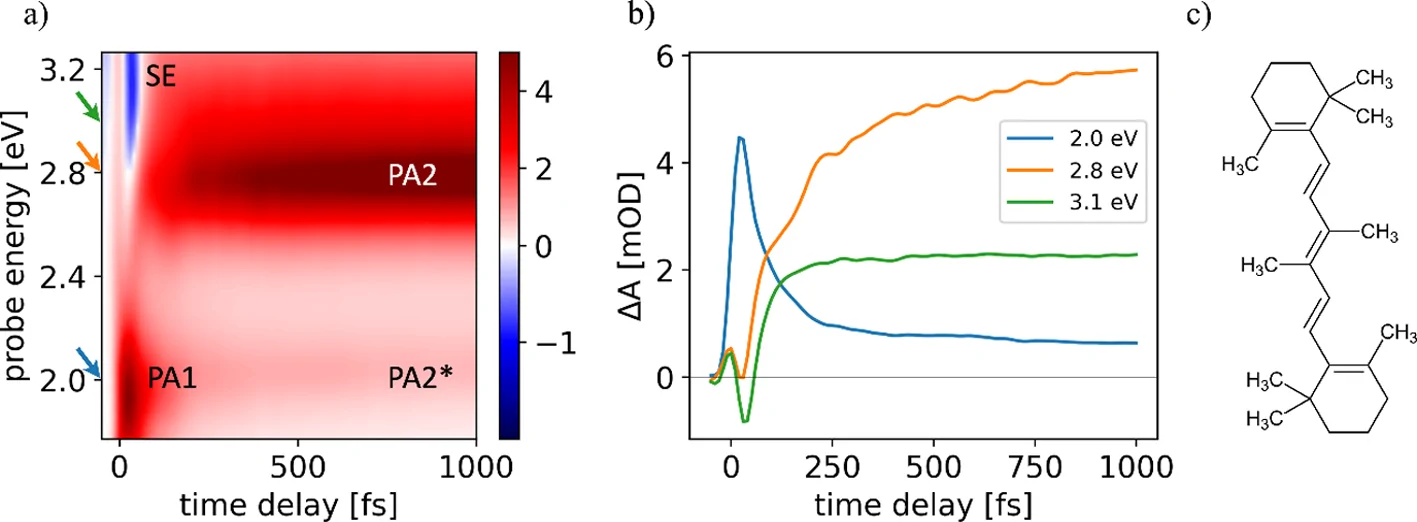
a) mini-5-*β*-carotene (m5) experimental TA map, from 0 to 1000 fs, in the 1.8–3.2 eV spectral window; b) cuts of the TA map at specific probe energies, namely at 2.0, 2.8, and 3.1 eV indicated by arrows on panel a), displaying the PA1 and SE disappearance from the initially pumped 1^1^ *B*_*u*_ state, and the PA2 dynamics from the later populated dark 2^1^ *A*_*g*_ state; c) chemical structure of the m5.

At longer delay times, the TA spectrum evolves into two long-lived positive bands: an intense feature centered at ∼2.75 eV (the second PA band, PA2) and a much weaker feature (PA2*) overlapping with the early-time PA1 region. Because the lifetimes of both bands far exceed the 1 ps experimental window, they are assigned as PA signals from the newly populated long-living dark 2^1^ *A*_*g*_ state.^40^ Figure 1b displays the TA dynamics at representative probe energies of 2.0, 2.8, and 3.1 eV. The temporal evolution at 2.0 eV captures the ultrafast decay of the 1^1^ *B*_*u*_ PA1 signal alongside the long-lived PA2* component, while the SE signal from the same initial state is clearly observed at 3.1 eV. While the 2^1^ *A*_*g*_ state is promptly populated via IC in ∼63 fs, the dynamics at 2.8 eV reveal that the PA2 band undergoes a subsequent, slower increasing peak intensity and spectral narrowing, rising with a time constant of 370 ± 10 fs as revealed by global analysis. As discussed below, this delay does not reflect the initial population of the 2^1^ *A*_*g*_ state, but rather a subsequent intra-state vibrational relaxation driven by the *β*-ionone rings.

Figure 2 compares experimental TA spectral cuts at selected time delays (panel a) with gas-phase TA spectra simulated at key critical structures and broadened using empirical Gaussian functions (panels b, d–e). For these critical structures, the corresponding bond-length alternation (BLA) and ring-torsion values are reported in panel c, together with a schematic representation of the BLA and torsional coordinates. To interpret the experimental transient absorption (TA) spectra, we assume a stepwise relaxation mechanism based on time scale separation, wherein rapid relaxation along the high-frequency BLA coordinate precedes slower planarization along the soft torsional mode. Upon excitation to the bright 1^1^ *B*_*u*_ state, initial rapid BLA relaxation brings the bright 1^1^ *B*_*u*_ state into near-degeneracy with the 2^1^ *A*_*g*_ state (gap of 0.01 eV at the 1^1^ *B*_*u*_ minimum, Table S1 in SI). This population transfer is further facilitated by the distribution of torsional angles already present at the FC geometry due to ground-state thermal fluctuations, which provides access to geometries favoring the 1^1^ *B*_*u*_ → 2^1^ *A*_*g*_ transition (see Section S3.3 in the SI for the relaxed torsional scan around the FC geometry and the corresponding population distribution at 300 K). The simulated TA spectra in Figure 2b directly validate this model and assign the primary experimental features. The TA spectra from the 1^1^ *B*_*u*_ minimum (orange curve) matches the earliest experimental features: a prominent PA band (PA1) and a SE signal. After IC, the relaxation proceeds on the 2^1^ *A*_*g*_ surface. To decouple the effect of BLA and torsional relaxation on 2^1^ *A*_*g*_ TA spectra, we consider a constrained reference snapshot where 2^1^ *A*_*g*_ is optimized but the torsional values are held to FC geometry (∼50°, green curve), which yields a broad PA2 band. Subsequent relaxation driven by the torsional motions toward the fully relaxed 2^1^ *A*_*g*_-min (cyan curve) causes the PA2 signal to grow in intensity, similarly the experimental trend seen in Figure 2a.

**Figure 2:**
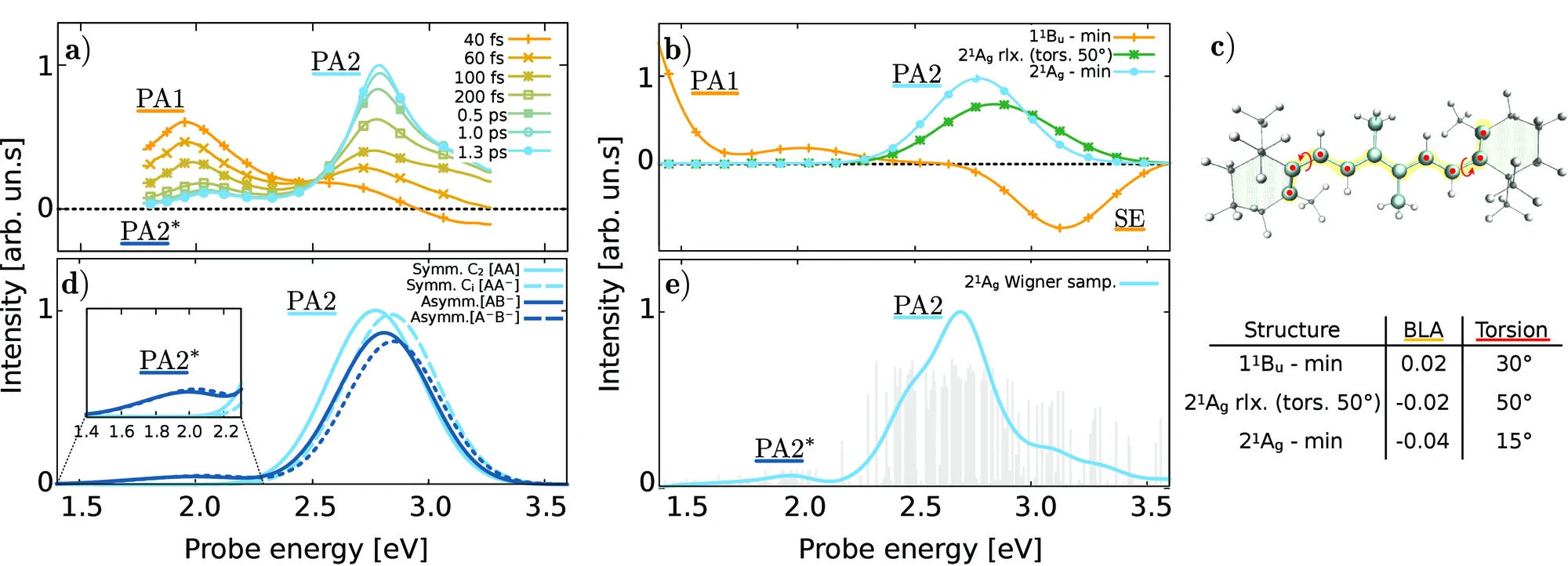
a) Experimental TA map cuts at selected time delays, displaying the PA1 band disappearance (from 1^1^ *B*_*u*_ state), and the PA2 appearance (from 2^1^ *A*_*g*_ state) and progressive narrowing in time; b) simulated TA signals from specific states/structures and c) corresponding BLA and torsion values: SE and PA1 from the 1^1^ *B*_*u*_ state minimum (orange curve), and PA2 from the 2^1^ *A*_*g*_ BLA relaxed structure with torsion kept fixed at their 50° FC values (green curve) and from its fully relaxed (planar) minimum (cyan curve). The PA2 signal narrowing is associated to the planarization of the *β*-ionone rings dihedral; d) simulated PA2 and PA2* from the 2^1^ *A*_*g*_ minima of 4 different m5 conformers, grouped as symmetric (cyan curve) and non-symmetric (blue curves): only the latter show the rise of a PA2* band as shown in the inset (see below for conformers labeling); e) same signals out of a Wigner sampling of 100 geometries at the 2^1^ *A*_*g*_ minimum (from optimization of the most stable - (C_2_) symmetric - m5 conformer), also showing the appearance of the PA2* band due to non-Condon effects arising from nuclear displacements around the optimized 2^1^ *A*_*g*_ minimum.

Beyond these main features, our calculations also clarify the origin of the weaker, low-energy PA2* band. As demonstrated in Figure 2d, ground-state conformational heterogeneity plays a key role in the emergence of this band. In m5, combinations of terminal-ring puckering phases and ring backbone torsion angles generate 16 thermally accessible conformers within 1 kcal/mol of the global minimum (Section S3.1,S3.2 of SI), split into symmetric (*C*_2_/*C*_*i*_) and non-symmetric geometries. While the symmetric conformers relax to equivalent ring-backbone dihedrals (∼15°), the non-symmetric conformers relax to asymmetric geometries with unequal dihedrals (∼10° and 20°). The simulated spectra on top of these non-symmetric 2^1^ *A*_*g*_ optimized minima uniquely exhibit the PA2* feature. Complementing this, Figure 2e accounts for dynamic nuclear fluctuations on the symmetric minimum via Wigner sampling at *T* = 300 K. The resulting ensemble spectrum captures coordinate-dependent transition dipole modulation, confirming that nuclear displacement along symmetry-breaking normal modes activates this feature even in symmetric structures via non-Condon effects. Across both non-symmetric optimized geometries and Wigner-displaced structures, the state *S*_*n*_ responsible for the weak PA2* band exhibits a multi-configurational character dominated by a mix of double (*H* ⇒ *L*), single (*H* → *L* + 1), and closed-shell ground-state configurations. Notably, at these same geomtries, the 1^1^ *B*_*u*_ → *S*_*n*_ transition is strongly allowed suggesting why symmetry-breaking dihedrals and non-Condon fluctuations efficiently impart dipole strength to the PA2* feature.

## The Role of the *β*-Ionone Rings Torsion in Car Photophysics

This dual-coordinate relaxation mechanism where a rapid BLA relaxation is followed by a slower, large-amplitude ring alignment with the backbone suggests a broader paradigm for the photophysics of Cars. Historically, a Car with a nominal conjugation length *N* is treated as a static polyenic chain. However, as illustrated by the model in Figure 3, our experimental and computational results for m5 reveal that the *β*-ionone ring–backbone torsion acts as a dynamic modulator of the effective conjugation length during photorelaxation: at the FC geometry, the molecule electronically resembles a shorter polyene, with an effective conjugation length approaching the *N* – 2 limit (corresponding to complete ring–backbone decoupling); as the rings rotate toward planarity on the 2^1^ *A*_*g*_ surface, conjugation progressively extends into the ring double bonds, ultimately approaching the electronic structure of a fully conjugated *N*-polyene at the 2^1^ *A*_*g*_ minimum. Naturally, the extent to which this mechanism influences photophysics depends on the overall chain length and the degree of ring planarization achieved. This effect is expected to be most significant for short Cars, where accounting for the change in the effective conjugation length has a stronger impact on the 2^1^ *A*_*g*_ energetic position and the molecule photo-relaxation. For medium-sized (*N* ≃ 7 – 9) Cars, inclusion of the effect of lateral rings should improve the description and lead to better quantitative agreement with experiments. In contrast, longer Cars are instead expected to be less affected, as the energy gap between the polyenes with *N* – 2 and *N* double-bonds becomes progressively narrower for increasing *N*.

**Figure 3:**
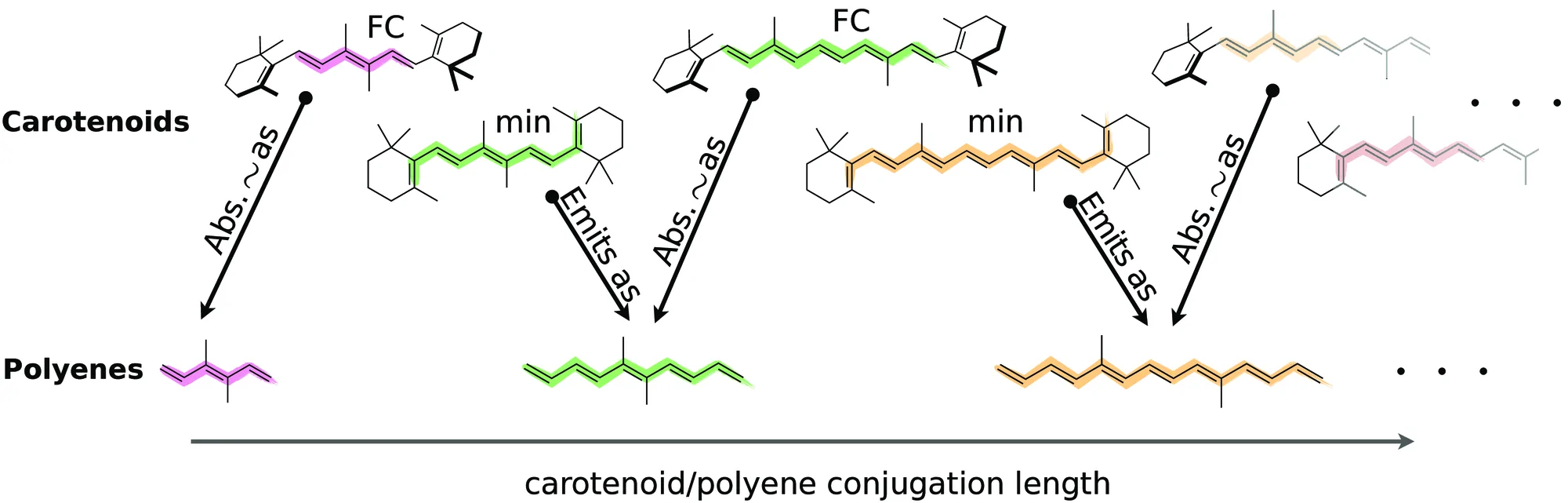
*β*-ionone rings as key tuning factors of Car photophysics. The Car-polyene analogy should be updated as prescribed here. In the schematic the similar conjugation lengths are highlighted with the same color. The photophysics of Car with conjugation length *N* is expected to interpolate between the photophysics of polyenes with conjugation length ∼*N* – 2 and *N* to capture absorption and emission data, respectively.

To test this hypothesis and quantify the energetic consequences of this interpolation, Figure 4a compares the calculated 2^1^ *A*_*g*_ energy profiles of m5 against those of the three- and five-double-bonded polyenes (p3 and p5, respectively). In the central panel, the *β*-ionone rings are held fixed at their FC dihedral angle (50°) relative to the backbone while only the BLA coordinate is allowed to relax. Under this constraint, the energy profile of m5 tracks the polyenic curves almost perfectly, behaving as a conjugated chain of intermediate length between p3 and p5. The side panels isolate the explicit effect of the torsional coordinate. When the rings are completely decoupled from the central polyenic chain by moving them to perpendicular 90° twist from FC (left panel), the energy of m5 converges to that of the shorter p3 polyene (at its FC). Conversely, at the quasi-planar 15° inter-ring dihedral geometry of the true 2^1^ *A*_*g*_ minimum (right panel), the energy drops to match the optimized 2^1^ *A*_*g*_-min of the longer p5 polyene.

**Figure 4:**
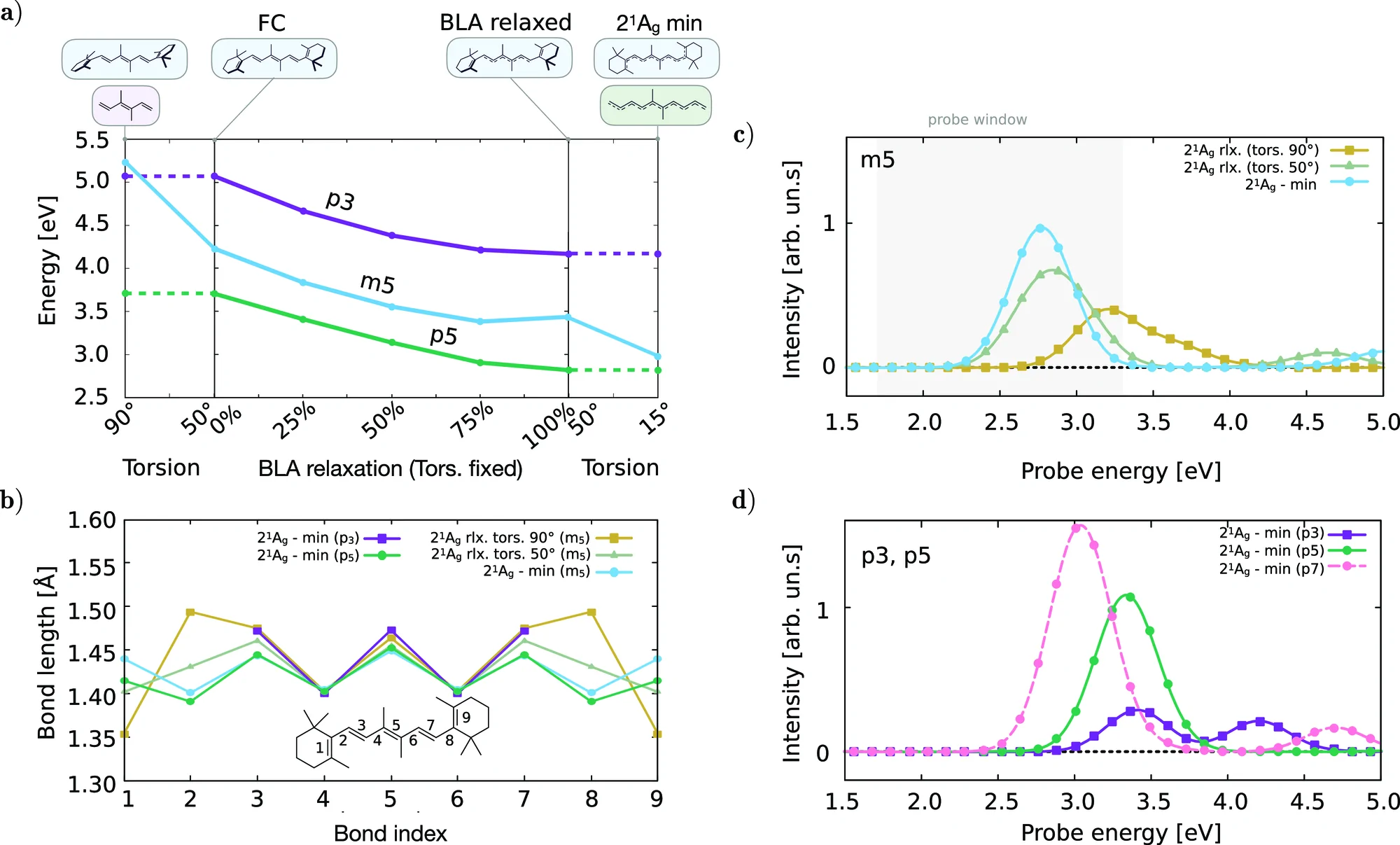
a) Energy scan from FC to 2^1^ *A*_*g*_-min decomposed along BLA and ring torsion coordinates for the 2^1^ *A*_*g*_ state of the m5 carotene and the p3 and p5 polyenes: along BLA (central panel), both the carotene and the two polyenes behave identically; ring torsion (lateral panels) is shown to tune the conjugation length: the m5 profile bridges between the p3 FC energy and the p5 2^1^ *A*_*g*_-min energy when the ring dihedrals are changed from 90° to 15°, respectively. Schematic structures are also reported at key points along the scan(s). b) C–C bond length at different optimized geometries of m5 and p3/p5 polyenes, highlighting that the torsional coordinate acts as a gate for complete relaxation of the BLA at the most lateral bonds; i.e., it only allows these bonds to relax and conjugate with the backbone when a sufficient planarization of the lateral rings is achieved. c) PA simulation from the 2^1^ *A*_*g*_ state at progressive planarization of the *β*-ionone torsion of m5; d) PA from the relaxed 2^1^ *A*_*g*_-min of polyenes p3, p5 and p7.

Crucially, a deeper analysis of the corresponding C–C bond-lengths (Figure 4b ) reveals that this torsional motion and the BLA relaxation are coupled. When the 2^1^ *A*_*g*_ state in m5 is optimized at the constrained 50° FC torsion, BLA relaxation occurs selectively along the central carbon chain (bonds 3-7), mirroring the interior profile of p5. However, because the rings remain largely out of plane the terminal bonds (bonds 1 and 9) cannot fully engage in conjugation and thus do not fully relax. It is only upon planarization to the 15° minimum that these terminal bonds are brought into full conjugation, allowing complete BLA relaxation on the 2^1^ *A*_*g*_ state. To visually emphasize this decoupling effect Figure 4b also shows when optimizing the 2^1^ *A*_*g*_ state with artificial 90° torsion which completely isolates the rings, yields a central BLA strictly matching the relaxed 2^1^ *A*_*g*_ state of shorter p3.

This coupled BLA–torsional relaxation dictates the evolution of the previously discussed PA2 transient spectroscopic features of m5, which are further analyzed here by comparison with the corresponding PA signals of p3 and p5. Figure 4c shows the TA spectra of m5 along the progressive planarization of the *β*-ionone torsional coordinate on the 2^1^ *A*_*g*_ state, while Figure 4d shows the corresponding spectra of the fully relaxed 2^1^ *A*_*g*_ states of the p3, p5 and p7 polyenes. While the shorter p3 polyene is characterized by a broad, double-peaked PA band, p5 shows a narrower and more intense PA band (a trend that is further confirmed by the additional evaluation of the p7 PA2 band). The spectral evolution of m5 captures this dynamic extension of conjugation length. When the rings are artificially decoupled at the 90°, the m5 spectrum closely mirrors the broad profile of the shorter p3. Allowing the central BLA chain to relax while keeping the rings frozen at the 50° FC torsional value produces an intermediate spectrum, more intense than p3 but broader and less intense than p5. Finally upon maximal alignment of rings at the fully relaxed minimum, the PA band shifts to its narrower and more intense profile. This spectral evolution demonstrates that the late-stage TA spectra changes reflect a concurrent torsionally-gated vibrational relaxation of the terminal bonds as the extended polyenic conjugation is fully established.

## Impact of Ground-State Conformer Heterogeneity on Car Spectroscopy and Dynamics

While the dynamic coupling of BLA and ring-backbone torsional coordinates describes the primary relaxation pathway, a complete picture of Car photophysics requires a comprehensive accounting of the ground-state conformational landscape. As introduced earlier, electronic structure calculations on m5 reveal multiple thermally accessible conformers within 1.0 kcal/mol of the *C*_2_-symmetric global minimum. This structural variation at FC is explicitly driven by the specific phase of the ring puckering in conjunction with the sign of torsion angle of the terminal rings with the central conjugated backbone. We assign A­(A^–^) and B­(B^–^) letters to represent different combination of these two independent coordinates (ring-torsion ± 50°, ring-puckering up/down as detailed in Section S3.1 of SI). This leads to 16 different conformations of the m5, resulting in a mixture of *C*_2_(AA,BB), *C*_*i*_(AA^–^,BB^–^) and non-symmetric (AB,AB^–^) geometries. (Note that these 16 structures reduce to 10 non-equivalent ones when removing those that can be superimposed by means of simple rotations. Note also that some of the structures are enantiomers, as, e.g., AB and B^‑^A^‑^, BB and B^‑^B^‑^, etc.)

Historically, these terminal-ring fluctuations have been treated as structural noise. However, recent studies on natural ketocarotenoids like canthaxanthin (CAN) show that *β*-ionone rings adopt energetically degenerate puckering states that dictate the barrier to ring rotation, creating distinct local minima on the ground-state potential energy surface.^41^ In m5, our detailed breakdown of 16 structures reveals a similar landscape. Because all these conformers display a similar magnitude of ± 50° ring torsion at FC, they exhibit nearly identical vertical energies at the FC point ( ∼3.54 eV for 1^1^ *B*_*u*_; ∼4.26 eV for 2^1^ *A*_*g*_, Table S8 in SI); however, their excited-state relaxation pathways diverge significantly. Specifically, symmetric conformers relax toward 2^1^ *A*_*g*_ minima with uniform ring torsions of approximately ± 15°, whereas non-symmetric conformers adopt asymmetric torsions of ∼10° and ∼20° at either ends. As already demonstrated, this structural asymmetry directly accounts for the emergence of the PA2* spectral feature, which remains absent in the symmetric 2^1^ *A*_*g*_-min structures. Furthermore, the calculated vertical emission energies from the 2^1^ *A*_*g*_-min vary significantly between AA, AB and BB conformers (1.9, 1.7, and 1.6 eV respectively, Table S9 of SI ), providing a physical basis for the red-shift in emission with increasing temperature observed in previous experimental works.^42^ Because AB and BB conformers lie higher in ground-state energy at 77 K the thermal population is dominated by the lowest-energy AA-type structures (∼90%). At 300 K, thermal activation also populates higher-energy conformers (∼40% AB and ∼10% BB), thus effectively lowering the average emission energy of the ensemble. More details of ground-state conformer energetics in Section S3.2 of SI.

Beyond steady-state features, our computations suggests how this ground-state heterogeneity might also affect excited-state lifetimes. The 1^1^ *B*_*u*_ – 2^1^ *A*_*g*_ energy gap at the 2^1^ *A*_*g*_ minimum varies across conformers from 0.5 to 0.9 eV (Table S9 in SI). This varying gap serves as an indicator of distinct 1^1^ *B*_*u*_ and 2^1^ *A*_*g*_ potential energy surface topologies among the *β*-ionone conformers that can lead to differing 1^1^ *B*_*u*_ → 2^1^ *A*_*g*_ internal conversion rates. In natural systems, such conformer-selective photophysics operates as a functional regulator; for instance, the Orange Carotenoid Protein exploits the ground-state conformational heterogeneity of CAN to partition energy relaxation pathways across multiple time scales,^43^ while LHCII utilizes conformational switches to control the transition between light-harvesting and photoprotection.^44^ Our findings show that m5, despite its short chain, captures essential intrinsic mechanisms that contribute to these complex relaxation pathways.

## Concluding Remarks

This work demonstrates that *β*-ionone ring torsion dictates the photophysics of (*β*-)­Cars by dynamically modulating their effective conjugation length. By directly combining ultrafast TA spectroscopy of the model m5-*β*-carotene with ab initio quantum-chemical computations that explicitly account for the *β*-ionone ring–backbone torsion, we show that this coordinate drives the molecule through a continuum of electronic structures—from an effectively shorter polyene at the FC geometry, with a conjugation length < *N*, to a fully conjugated *N*-polyene at the 2^1^ *A*_*g*_ minimum. This structural evolution occurs on a ∼370 fs time scale in m5 and is directly encoded in the measured TA signatures through progressive spectral narrowing and intensity enhancement of the PA2 band. Thus, we demonstrate that the conventional practice of mapping Car photophysics onto a single, static polyene neglects this dynamic structural transformation.

Additionally, our conformational analysis identified a landscape of symmetric and non-symmetric (m5) structures that are thermally accessible in the ground state. These structures exhibit distinct 1^1^B_*u*_–2^1^A_*g*_ energy gaps at the 2^1^A_*g*_-minimum, and are therefore expected to undergo photoinduced relaxation on different time scales. Moreover, non-symmetric structures display a weak photo-absorption band (labeled PA2* in this work) that is absent in their fully symmetric counterparts. Finally, our calculations predict distinct emission energies for these structures, suggesting that changes in their relative populations between 77 and 300 K underlie the temperature-dependent emission reported previously.

The magnitude of the ring-backbone torsional effect will naturally scale with chain length: most pronounced for short Cars where a two-double-bond change represents a large fractional alteration in conjugation, remains significant for medium-length systems (*N* ≈ 7-9) where quantitative spectral predictions require explicit treatment of ring dynamics, and diminishes asymptotically for very long chains where *N* – 2 and *N* polyene energetics converge. This might have relevant implications for Cars in conformationally constrained environments, and suggests novel insight into long-standing questions regarding intermediate dark states, multi-exponential decay kinetics, and chain-length dependencies observed across the Car family. Future experimental studies on longer Cars with explicit ring-torsion resolution will be necessary to validate the broader applicability of this dynamic conjugation-tuning mechanism in biological light-harvesting systems and photoprotective processes.

## Methods

### Computational Methods

The electronic structure of the m5 Car, as well as those of the p3 and p5 polyenes, were evaluated in gas-phase at the multireference CASPT2 level of theory with the quantum chemistry package OpenMolcas.^45,46^ Geometry optimizations used the in-house developed (freely available) code COBRAMM^47^ which allows to use the robust optimizer of Gaussian16.^48^ All CASSCF/CASPT2 computations made use of a mixed basis-set with conjugated carbon atoms treated at the cc-pVDZ level and the remaining atoms (C/H for m5, only H for p3/p5/p7) at the ANO-S-VDZ level to reduce the computational cost. CASPT2 calculations were performed on top of a state-averaged CASSCF reference with the full valence *π*/π* system in the CASSCF active-space. For m5 and its corresponding polyene p5, a CAS­(10,10) active space consisting of the full *π*-system (5*π* and 5π*-type orbitals) and a total of 10 active electrons was employed. For p3, a smaller CAS­(6,6) full-*π* active space was used instead.

The CASSCF results were then improved via the rotated multi-state flavor of CASPT2 (RMS-CASPT2),^49,50^ ensuring an accurate description of potential energy surfaces. RMS-CASPT2 in fact provides a balanced description of the states energetics both near and far from electronic states crossings, setting the IPEA and IMAG parameters to 0.0 and 0.2 hartree, respectively.^51,52^ We refer to this level as SAx-CAS­(10,10)/RMS-CASPT2, where x is the number of states considered in the state-average (SA) procedure for the given calculation. Since the 1^1^ *B*_*u*_ state is higher in energy at CAS level and only becomes lower after CASPT2 correction, an adequate number of states was included in the state-averaging procedure.

We note that all calculations were performed in the gas phase, whereas the experiments were conducted in cyclohexane. Nonetheless, the latter is a nonpolar solvent and is therefore expected to have only a minor impact on the electronic structure of m5. Our gas-phase calculations are thus well suited to reproduce the experimental conditions while maintaining a reasonable computational cost.

Three main types of computations were performed: (i) geometry optimization of the critical points; (ii) geometry scans along the key relaxation coordinates; (iii) single-point and TA calculations on specific geometries. Additional details are reported in the Supporting Information.

**i) Geometry optimization.** The molecular ground-state for all m5 conformers and p3, p5 polyenes was optimized at the MP2/cc-pVDZ level of theory. The excited-state manifolds were treated according to the specific electronic nature of the target states:

- **The dark** **2** ^**1**^ ***A***_***g***_ **state:** This state is predominantly covalent in character and is well-described by static correlation.^53,54^ Our analysis shows that the CASSCF wavefunction is qualitatively robust for this state; it remains the second root and its character is largely preserved upon the inclusion of perturbative corrections.
  Therefore, the 2^1^ *A*_*g*_ minima were optimized at the SA2-CAS­(10,10) level. A similar framework was also applied to polyenes for the 2^1^ *A*_*g*_ minima optimization using SA3-CAS­(10,10) and SA3-CAS­(6,6) for p5 and p3, respectively. For p7 a SA3-CAS­(14,14) level was employed.
- **The bright** **1** ^**1**^ ***B***_***u***_ **state:** In contrast, the 1^1^ *B*_*u*_ state possesses significant ionic character and is poorly described at the CASSCF level.^55^ Without dynamic correlation, this state appears as a high-lying root (e.g., root 6) with a heavily mixed and fragmented configuration. To recover the correct energetic ordering and *unmix* the state character, the inclusion of dynamic correlation is mandatory. Consequently, the 1^1^ *B*_*u*_ state was optimized at the RMS-CASPT2 level. For the m5 conformers, this was performed using an SA3-CAS­(4,4)/RMS-CASPT2 scheme, employing a reduced active space focused on the essential frontier orbitals to accurately resolve the state.

**ii) Geometry scans along key relaxation coordinates.** Geometry scans along the BLA and *β*-ionone rings torsion allow to decompose the relaxation pathway from the FC geometry to the 2^1^ *A*_*g*_ minimum. These scans were conducted using a geodesic interpolation approach.^56^ The energies in these calculations were computed at SA8-CAS­(10,10)/RMS-CASPT2 for m5 and p5, and at SA4-CAS­(6,6)/RMS-CASPT2 for p3.

**iii) Single-point & TA calculations.** The single-point computations for the energetics of ground-state, 2^1^ *A*_*g*_ and 1^1^ *B*_*u*_ for m5 at various critical structures were performed at RMS-CASPT2 level, utilizing SA8-CAS­(10,10) as the underlying CASSCF wavefunctions. The electronic structure at key geometries (FC and 2^1^ *A*_*g*_-min) of the 10 non-equivalent conformers are reported in Tables S8–S10 of the SI.

A higher number of states were used in state-averaging procedure to describe two manifolds of states interrogated by the probe pulse in TA. These computations were done at SA40-CAS­(10,10)/state-specific (SS-)­CASPT2 level employing only a state-specific CASPT2 correction of underlying 40 CASSCF states. The transition dipole moments between pair of states were evaluated using the RASSI module of OpenMolcas. The Wigner sampling of geometries (100 structures sampled at a temperature of *T* = 300 K) at the 2^1^ *A*_*g*_ minimum were generated using CASSCF normal modes on top of the CASSCF optimized 2^1^ *A*_*g*_ state minimum. Due to the large number of calculations required in this case, a state average of 20 was used for the TA calculations (SA40-CAS­(10,10)/SS-CASPT2 level).

For the polyenes TA spectra (reported in Fig. 4) the following levels of theory were employed SA12-CAS­(6,6)/SS-CASPT2, SA20-CAS­(10,10)/SS-CASPT2 and SA30-CAS­(14,14)/SS-CASPT2 for p3, p5 and p7, respectively.

### Experimental TA Setup

Ultrafast TA measurements were performed using a home-built experimental system based on an amplified Ti:sapphire laser (Coherent Libra), delivering ∼100 fs pulses centered at 800 nm with a 1 kHz repetition rate.^57^ A portion of the laser output was frequency doubled and used to pump a non-collinear optical parametric amplifier (NOPA), producing broadband visible pulses, which were temporally compressed by means of chirped dielectric mirrors. These compressed pulses were then frequency doubled in a 20-*μ*m-thick *β*-barium borate (BBO) crystal to generate UV pump pulses, tunable across the 250–300 nm spectral region. The UV pump pulse was tuned at 294 nm and compressed with a pair of prisms down to a duration of 24 fs.

Broadband probe pulses covering the 320–650 nm spectral range were produced through white-light continuum generation by tightly focusing a fraction of the fundamental laser output onto a 2 mm-thick CaF_2_ plate. The pump and probe beams were non-collinearly overlapped at the sample position, setting the relative polarization to the magic angle (54.7°), to eliminate the effects of rotational anisotropy. The sample was placed in a 1 mm-thick quartz cuvette. The transmitted probe was recorded by a spectrometer operating at the laser repetition rate. The TA signal was obtained by varying the pump-probe delay using a computer-controlled optical delay line. The probe spectrum was measured with and without the pump pulse, which was modulated using a mechanical chopper running at 500 Hz.

### Sample Preparation and m5 Synthesis

Mini-5-*β*-carotene (m5) was synthesized as follows. *β*-Ionone (4-(2,6,6-trimethylcyclohexenyl)-3-buten-2-one, C_13_ ketone) was purified by silica gel chromatography (diethyl ether/*n*-hexane = 1:9). McMurry coupling of the C_13_ ketone, mediated by low-valent titanium generated *in situ* from TiCl_4_,^58–60^ afforded m5. The product was recrystallized from hexane or benzene to give analytically pure material. The all-trans configuration was confirmed by ^1^H NMR spectroscopy (JEOL JNM-LA400).

## Supplementary Material



## Notes

This pdf contains m5 structure and relevant coordinates, conformational analysis and additional details about electronic structure computations. (PDF)
