# Supplementary material for: How β‑Ionone Ring Dynamics and Conformational Heterogeneity Control Carotenoid Photophysics
Source: J Phys Chem Lett. 2026 Sep 17;17(39):11171–9. doi: 10.1021/acs.jpclett.6c02098 (PMC13637073; doi:10.1021/acs.jpclett.6c02098)
Supplement: Supplementary file 1 [file jz6c02098_si_001.pdf]

SUPPORTING INFORMATION FOR

How β-Ionone Ring Dynamics and Conformational Heterogeneity Control Carotenoid Photophysics

V. K. Jaiswal,[*,†,‖] L. Uboldi,[‡,‖] A. Bonvicini,[†,¶,‖] A. Nenov,[†] M. Garavelli,[†] L. Muccioli,[†] D. Polli,[‡] H. Hashimoto,[§] Chiasa Uragami,[§] G. Cerullo,[*,‡] and F. Segatta[*,†]

†*Dipartimento di Chimica Industriale "Toso Montanari", Università di Bologna - Alma Mater Studiorum, Via Piero Gobetti 85, 40129 - Bologna, Italy*

‡*Dipartimento di Fisica, Politecnico di Milano, Piazza Leonardo da Vinci 32, I-20133, Milano, Italy*

¶*Theoretical Chemistry Laboratory, Unit of Theoretical and Structural Physical Chemistry, Namur Institute of Structured Matter, University of Namur, B-5000 Namur, Belgium*

§*Department of Applied Chemistry for Environment, Graduate School of Science and Technology, Kwansei Gakuin University, 1 Gakuen-Uegahara, Sanda 669-1330, Japan*

‖*These authors contributed equally to this work.*

E-mail: vishalkumar.jaiswal3@unibo.it; giulio.cerullo@polimi.it; francesco.segatta@unibo.it

This PDF includes:

- m5 structure and relevant coordinates;
- Conformational analysis;
- Electronic Structure: additional details

S1 Experimental data

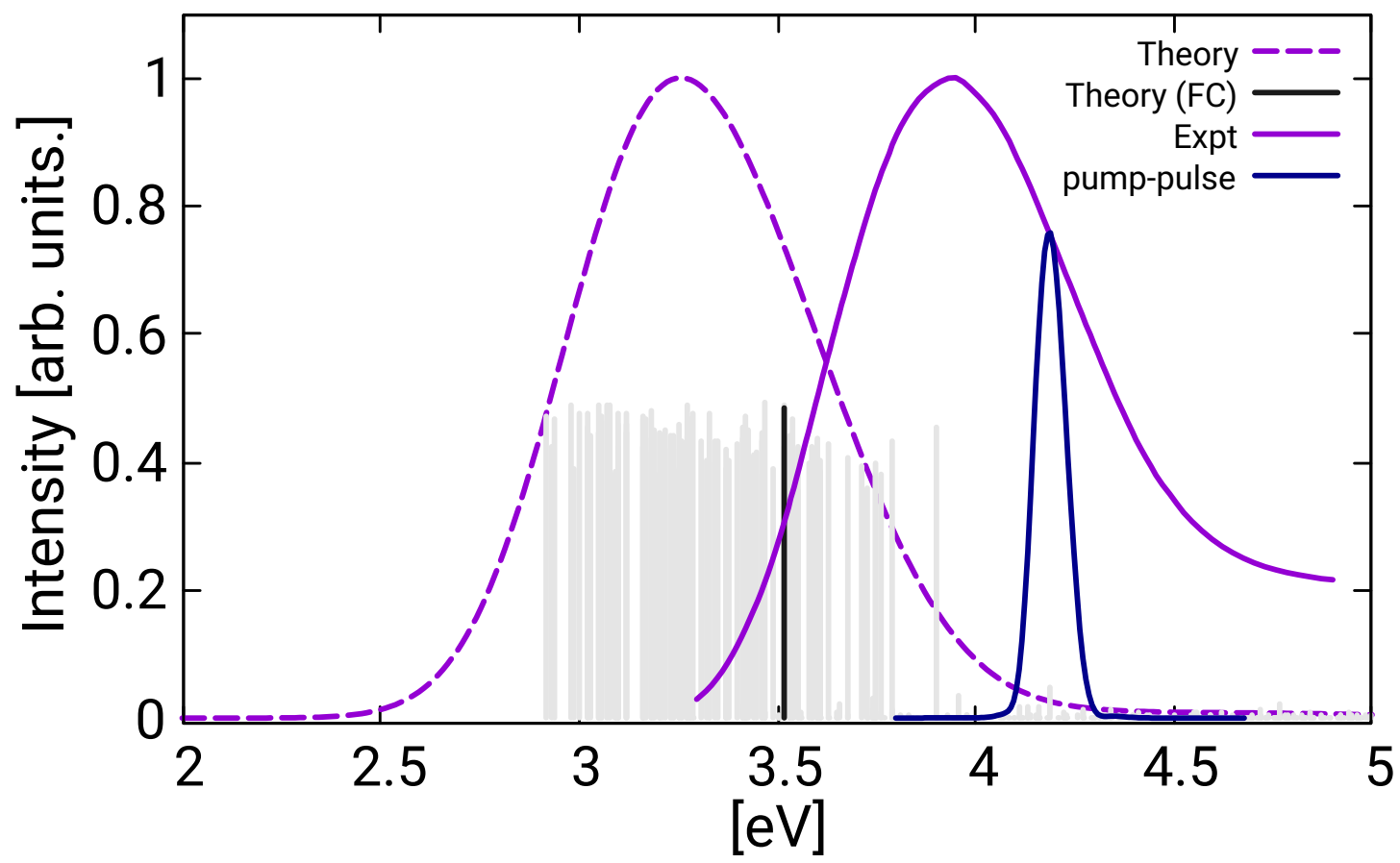

Figure S1: Experimenal absorption spectra of m5 in cyclohexane alongwith the pump-pulse. Also shown in simulated spectra computed on Wigner ensemble of the most stable conformer.

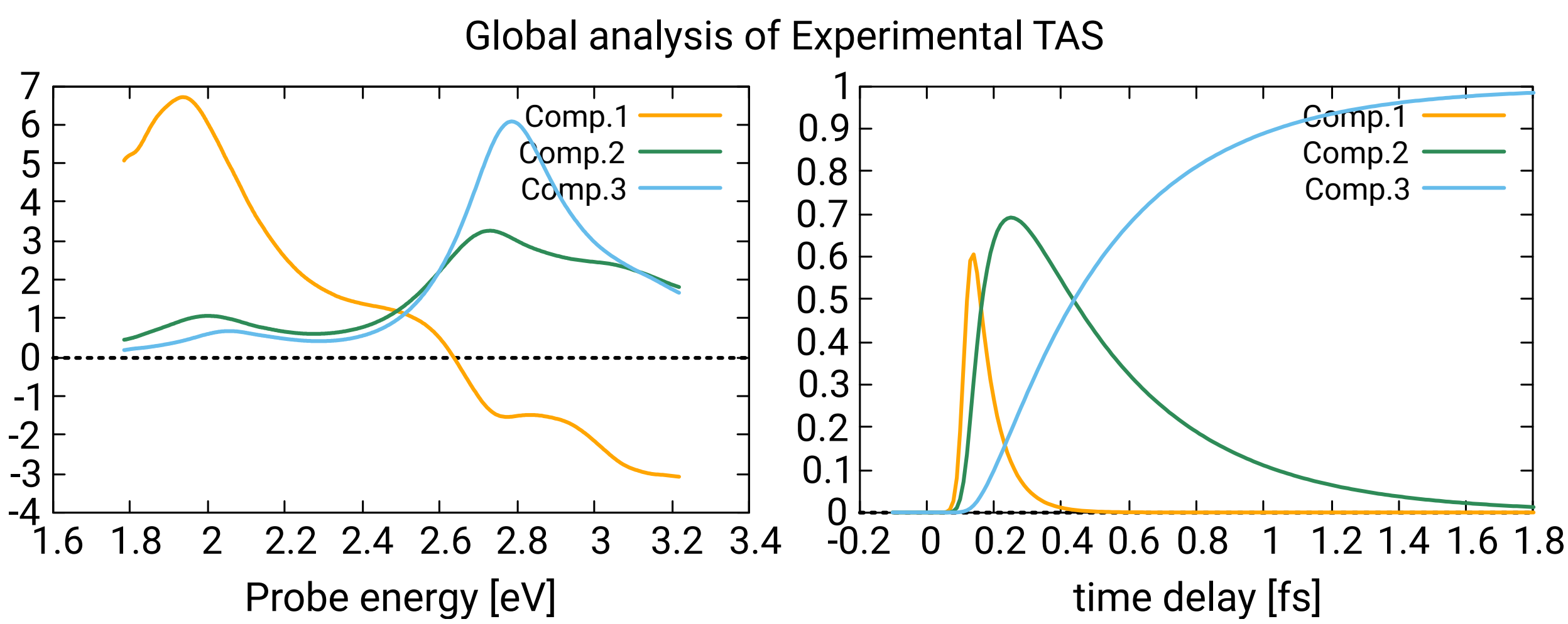

Figure S2: Evolution associated spectra (EAS) (left) and associated time-evolution obtained by global analysis of the experimental spectra in Figure 1 of main text.

S2 m5 structure and relevant coordinates

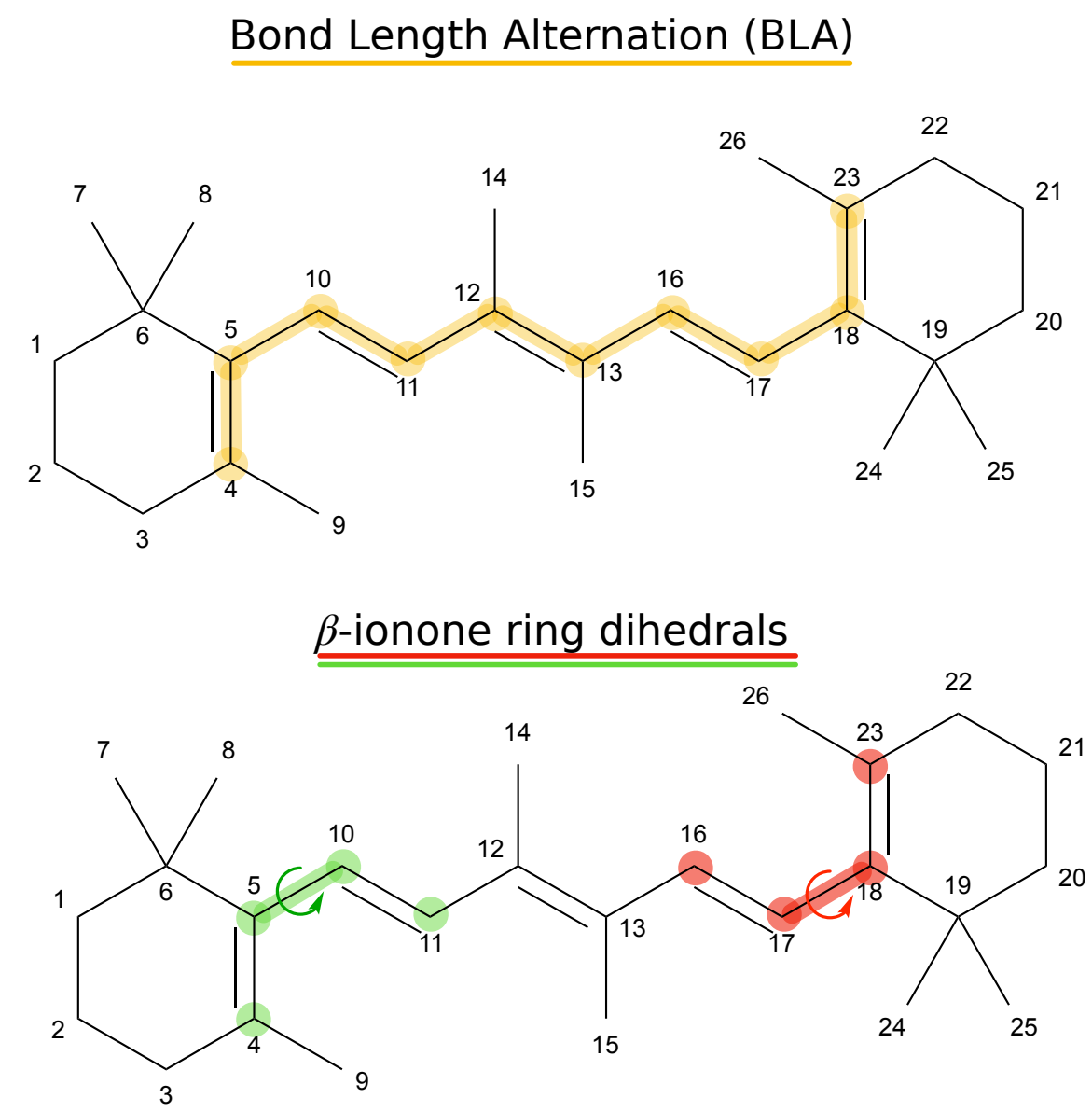

Figure S3: m5 molecular structure, where the bond length alternation (BLA) and β-ionone ring torsional coordinates are also highlighted. BLA is defined as as the average difference between the bond lengths of adjacent single (C−C) bonds and double (C=C) bonds. BLA is large when single and double bonds are markedly different, and tends to zero when they become similar in length.

S3 Conformational analysis

S3.1 Coordinates and nomenclature

As discussed in the main text, we labeled the different conformers with two letters indicating the configurations of the respective (left/right) rings: A (corresponding to a −50° dihedral and up-pointing puckering), B (−50°/down), and their inverse A^- (+50°/down) and B^- (+50°/up). A and B rings conformations present a different interaction between the rings and the chain, as highlighted in Figure S4.

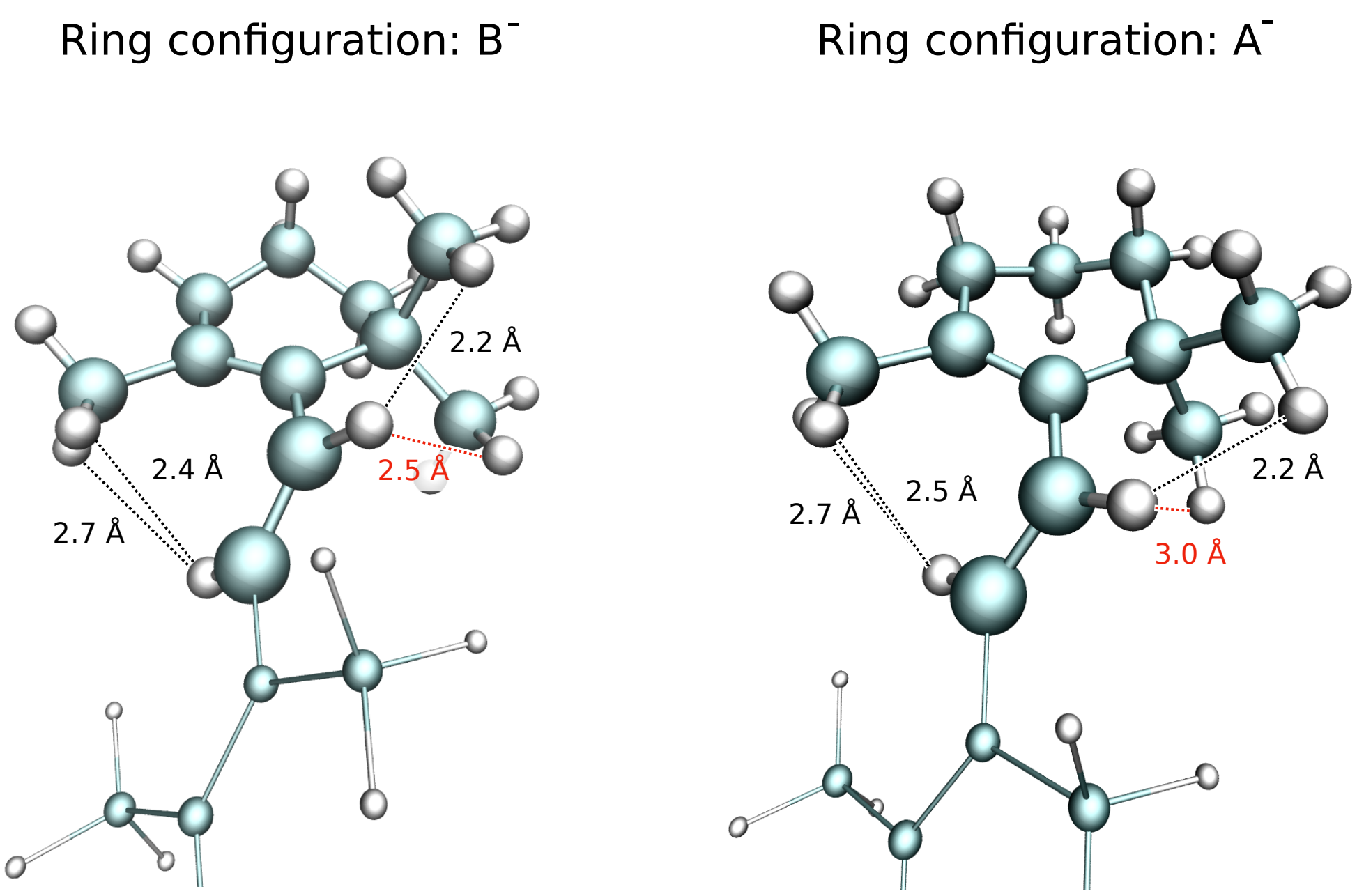

Figure S4: This figure highlights the ring-chain interaction that determine the conformer energy. The B^- and A^- conformers are represented (+50°/up-pointing puckering and +50°/down-pointing puckering, respectively). As detailed below, 0.45 kcal/mol energy penalty has to be taken into account for B/B^- rings configurations, as this ring conformation displays smaller H(ring)-H(chain) distances.

A conformational analysis of m5 that identified 16 thermally accessible structures within 1.0 kcal/mol of the global minimum (at the MP2/cc-pVDZ level of theory). As organized in Figure S5, these conformers are defined by combinations of ring torsion signs (±50°) and puckering directions (up/down), resulting in a mixture of C_2, C_i, and non-symmetric

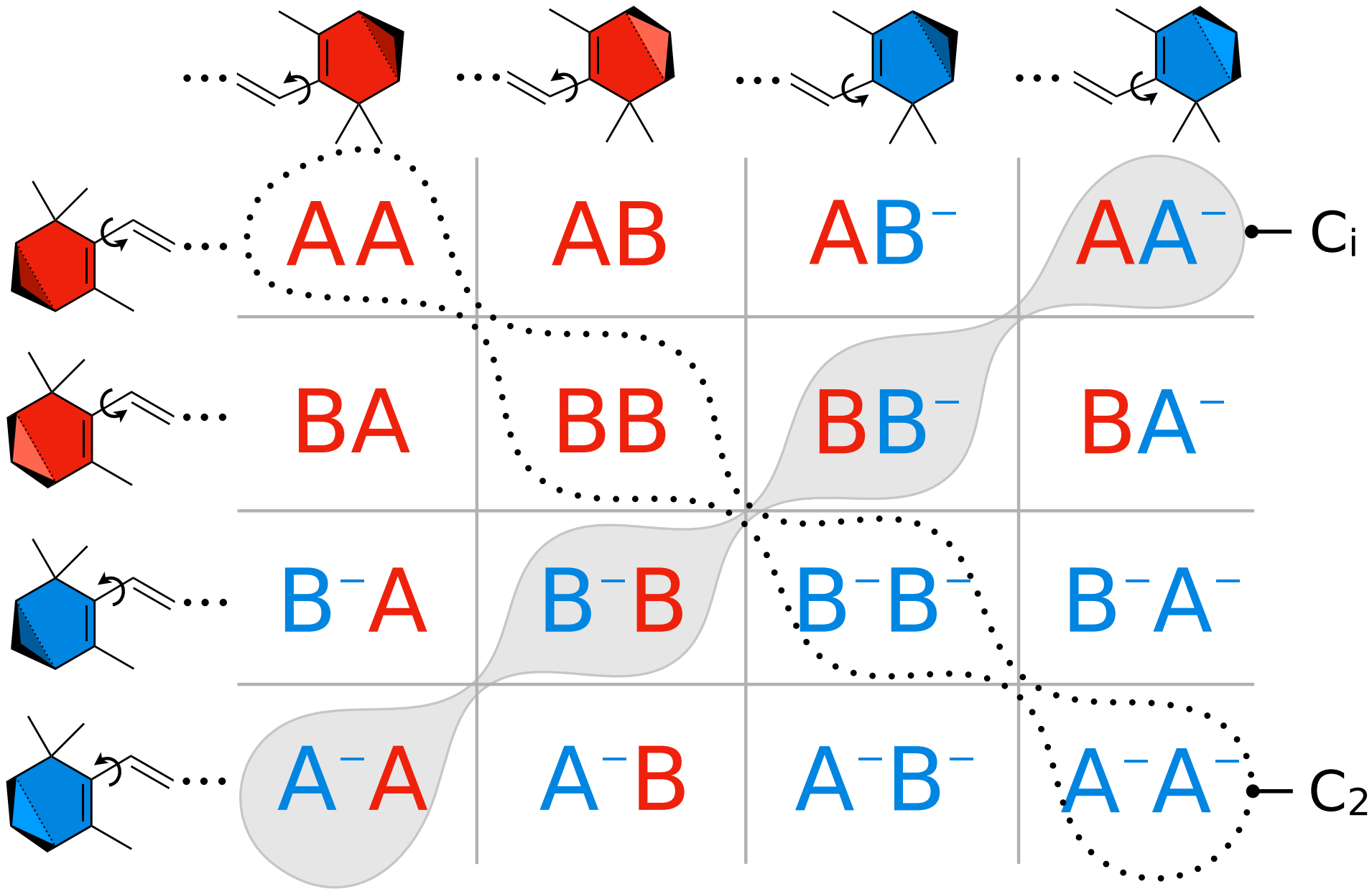

Figure S5: The 16 m5 conformers organized according to: i) the value of the rings torsion (+50° in blue, and −50° in red) and ii) the phase of the puckering (pointing up- or downwards). We labeled the different conformers with two letters indicating the configurations of the respective (left/right) rings: A (corresponding to a −50° dihedral and up-pointing puckering), B (−50°/down), and their inverse A^- (+50°/down) and B^- (+50°/up). Notably, each B or B^- ring configuration presents a 0.45 kcal/mol energy penalty (with respect to A or A^-) due to an unfavorable interaction between one of the ring methyl-groups and the Car backbone (see Section S2 in the supporting information). The same table highlights the symmetric (C_2 on the diagonal and C_i in the anti-diagonal) and non-symmetric conformers (the remaining off diagonal elements).

geometries.[1] While these structures exhibit nearly identical vertical energies at the FC point (due to the the fact that all structures display a ±50° ring torsion), their excited state relaxation pathways diverge significantly. Specifically, symmetric conformers relax toward 2^1A_g minima with uniform ring torsions of approximately ±18°, whereas non-symmetric conformers adopt asymmetric torsions of ∼10° and ∼20° at either ends.

[1]Note that these 16 structures reduce to 10 non-equivalent ones when removing those that can be superimposed by means of simple rotations. Note also that some of the structures are enantiomers, as, e.g., AB and B^-A^-, BB and B^-B^-, etc.

S3.2 Conformers energetics

The 16 conformers structures can be organized in a three-level energy ladder, where each consecutive level increases the energy by about 0.45 kcal/mol due to the unfavorable interaction (closer proximity) between one of the chain H atoms and the nearby ring methyl group: therefore, on the lowest level lie 4 structures with no unfavorable interactions (AA, AA^-, A^-A^-), on the middle level 8 structures in which the unfavorable interaction is only present on one of the two rings (AB, AB^-, B^-A, A^-B^-), and the upper level presents 4 structures with unfavorable interactions at both rings sides (BB, BB^-, B^-B^-). See Figure S6. Within the introduced notation, each "B" in the conformer label highlight a 0.45 kcal/mol energy penalty. Note also that all the non-symmetric conformers lie in the second energy level of the ladder.

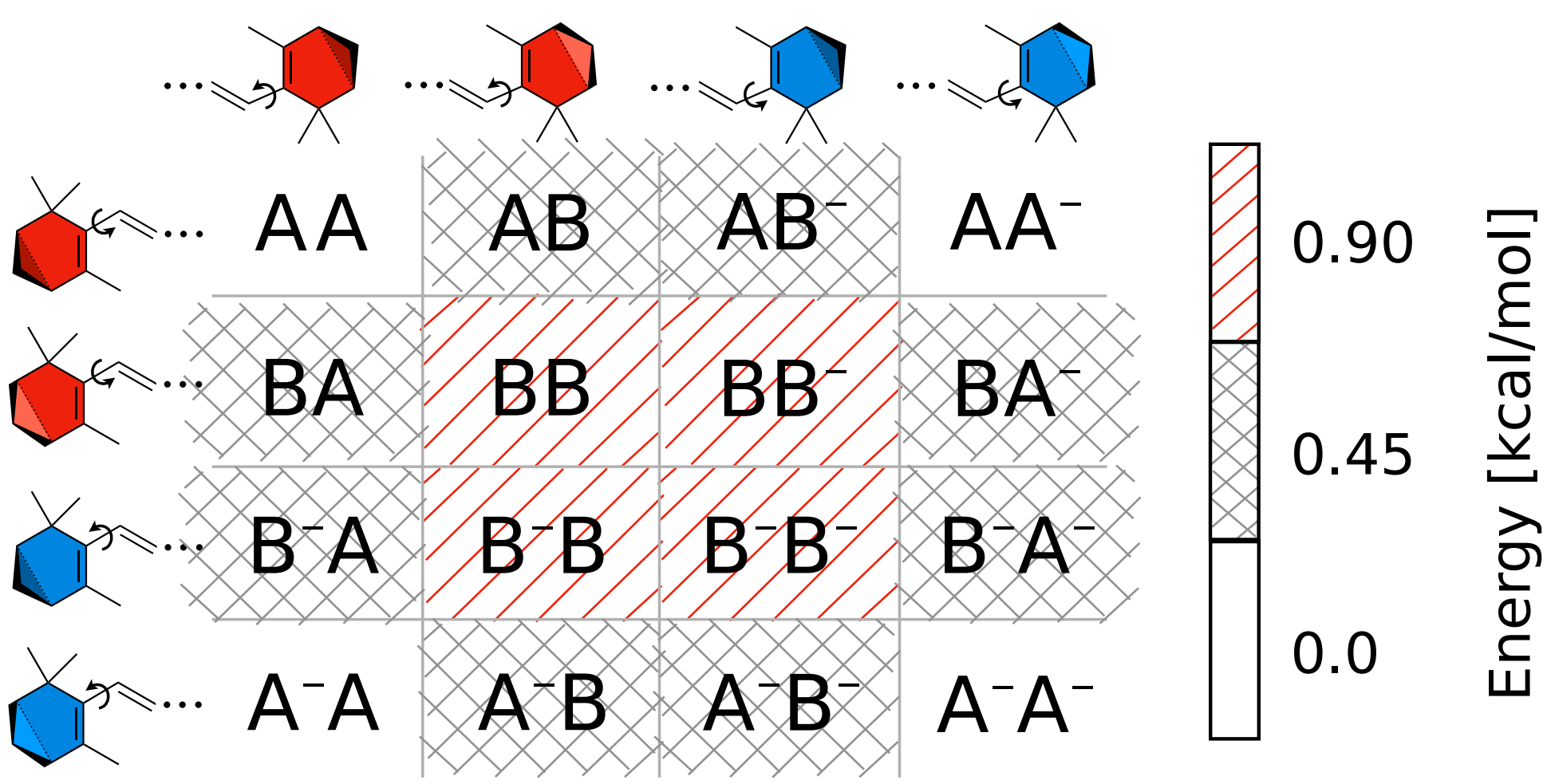

Figure S6: Same schematic of the main text Figure 5, in which the energy of the various conformers is highlighted (first, second and third energy levels are depicted with white, gray and red backgrounds, respectively).

Taking into account the thermal population at a given temperature T, given by the Boltzmann formula

$$p_i = \frac{g_i e^{-E_i/RT}}{\sum_j g_j e^{-E_j/RT}} \tag{S1}$$

where p_i g_i and E_i are the population, the degeneracy and the energy of the i-th level in the conformer energetic ladder, one finds that, at 77 K and 300 K, the thermal population of the three levels is given by:

	level 1	level 2	level 3
Degeneracy	4	8	4
T = 77 K	90.2%	9.5%	0.3%
T = 300 K	46.3%	43.5%	10.2%

S3.3 Distribution of β-ionone torsions at Franck-Condon region at room temperature

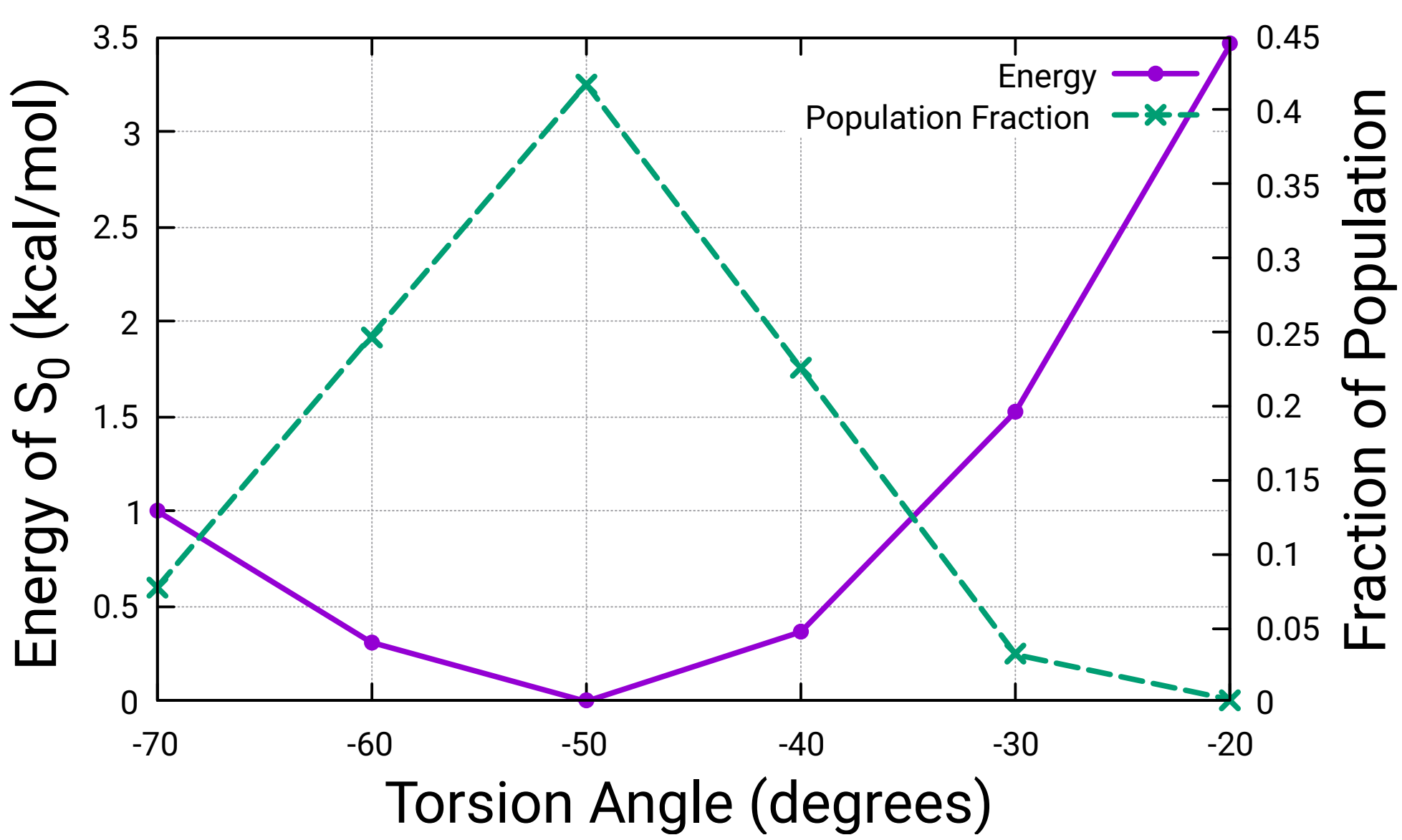

Figure S7: Relaxed scan at MP2/Cc-PVDZ level of the β-ionone torsions. Energies in kcal/mol relative to the FC and associated population fraction (at 300K) are shown.

S4 Electronic Structure: additional details

This section contains:

- The m5 active space;
- Tables with the electronic structure data out of the CASSCF/CASPT2 calculations.
- The quantum chemical rationale behind the the PA2 band narrowing in time (i.e., along the m5 planarization).

m5 Active Space

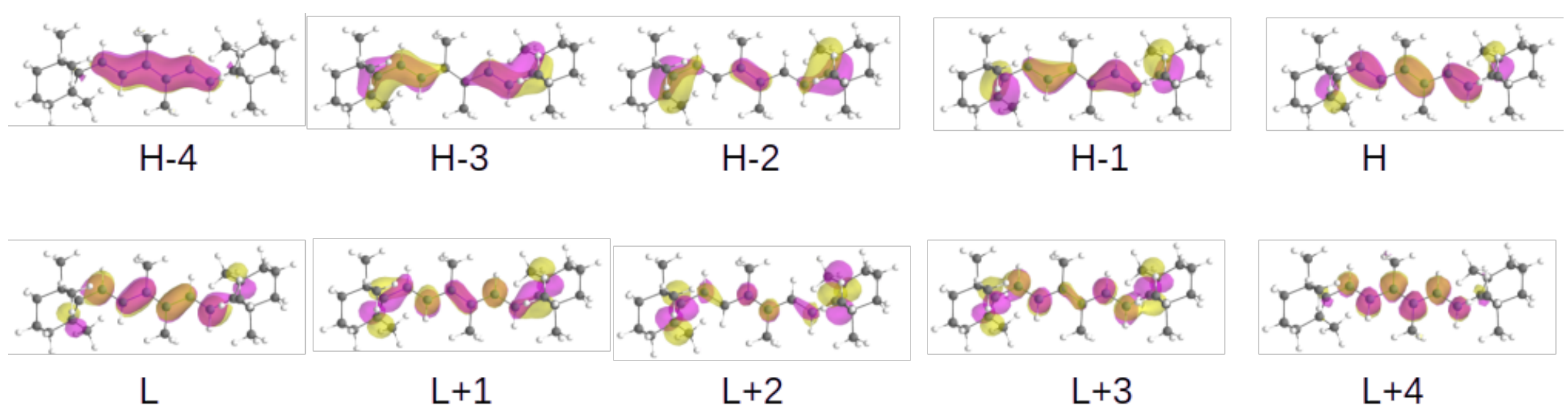

Figure S8: Active-space orbitals of m5 mini-carotene used in the CASSCF/CASPT2 calculations.

Electronic structure data

Tables S1-S7 refer to calculations performed on the AA m5 conformer. Tables S8-S10 refers to calculations performed on the different m5 conformers.

Table S1: Energies (in eV) of lowest singlet states at critical m5 geometries (SA8-CAS(10,10)/RMS-CASPT2).

State	FC	1^1B_u-min	2^1A_g-min
GS	0	0.44	1.13
1^1B_u	3.52	3.17	3.54
2^1A_g	4.23	3.18	3.06

Table S2: Energies (in eV) of the lowest singlet states at critical geometries of the polyene p5 (SA8-CAS(10,10)/RMS-CASPT2).

State	FC	2^1A_g-min
GS	0	0.56
1^1B_u	3.70	3.65
2^1A_g	3.71	2.82

Table S3: Energies (in eV) of the lowest singlet states at critical geometries of the polyene p3 (SA4-CAS(6,6)/RMS-CASPT2).

State	FC	2^1A_g-min
GS	0	0.77
1^1B_u	4.68	4.17
2^1A_g	5.07	4.66

Table S4: Major electronic configurations of the lowest singlet states of the m5 at Franck-Condon and 2^1A_g minimum. (SA8-CAS(10,10)/RMS-CASPT2).

	Electronic configuration (weight)	
State	FC	2^1A_g-min
1^1B_u	$H \rightarrow L$ (0.78)	$H \rightarrow L$ (0.71)
2^1A_g	$H \Rightarrow L$ (0.28)<br>$H-1 \rightarrow L$ (0.18)<br>$H \rightarrow L+1$ (0.10)	$H \Rightarrow L$ (0.25)<br>$H-1 \rightarrow L$ (0.19)<br>$H \rightarrow L+1$ (0.15)

Table S5: Major electronic configurations of the lowest singlet states of the polyene p5 at Franck-Condon and 2^1A_g minimum. (SA8-CAS(10,10)/RMS-CASPT2).

	Electronic configuration (weight)	
State	FC	2^1A_g-min
1^1B_u	$H \rightarrow L$ (0.77)	$H \rightarrow L$ (0.71)
2^1A_g	$H \Rightarrow L$ (0.29)<br>$H-1 \rightarrow L$ (0.17)<br>$H \rightarrow L+1$ (0.13)	$H \Rightarrow L$ (0.26)<br>$H-1 \rightarrow L$ (0.19)<br>$H \rightarrow L+1$ (0.16)

Table S6: Major electronic configurations of the lowest singlet states of the polyene p3 at Franck-Condon and 2^1A_g minimum. (SA4-CAS(6,6)/RMS-CASPT2).

	Electronic configuration (weight)	
State	FC	2^1A_g-min
1^1B_u	$H \rightarrow L$ (0.87)	$H \rightarrow L$ (0.77)
2^1A_g	$H \Rightarrow L$ (0.30)<br>$H-1 \rightarrow L$ (0.24)<br>$H \rightarrow L+1$ (0.15)	$H \Rightarrow L$ (0.30)<br>$H-1 \rightarrow L$ (0.22)<br>$H \rightarrow L+1$ (0.15)

Table S7: Transitions giving rise to the main PA signals recorded in transient absorption. Transition labeling (from the low-lying excited states of interest - 1^1B_u, 2^1A_g - to the bright higher-lying states responsible of the PA signals - labeled as S_a and S_b), spectral labeling, transition energy (in eV), oscillator strength, main configurations of the arrival states (SA20-CAS(10,10)/SS-CASPT2).

Transition	Spectral Label	ΔE (eV)	f	S_x elect. config. (weight)
$1^1B_u \rightarrow S_a$ (at 1^1B_u-min)	PA1	1.59	1.54	$H \rightarrow L+1$ (0.27)
				$H \Longrightarrow L$ (0.20)
$2^1A_g \rightarrow S_b$ (at 2^1A_g-min)	PA2	2.77	0.72	$H-1 \rightarrow L+1$ (0.25)
				$H-2 \rightarrow L$ (0.12)
$2^1A_g \rightarrow S_a$ (at 2^1A_g-min)	PA2*	1.87	0	$H \rightarrow L+1$ (0.25)
				$H \Longrightarrow L$ (0.12)
$\langle 2^1A_g \rightarrow S_b \rangle_{\text{Wigner}}$	PA2	2.68	0.46	
$\langle 2^1A_g \rightarrow S_a \rangle_{\text{Wigner}}$	PA2*	1.88	0.02	

* * *

Table S8: Absorption energies (in eV) at Franck-Condon geometry for all the (non-equivalent) m5 conformers (SA8-CAS(10,10)/RMS-CASPT2).

	**C_2**				**C_i**		**C_1**			
	AA	BB	B^-B^-	A^-A^-	AA^-	BB^-	AB^-	A^-B^-	AB	A^-B
1^1B_u	3.52	3.55	3.55	3.52	3.54	3.57	3.55	3.54	3.54	3.55
2^1A_g	4.23	4.27	4.27	4.23	4.25	4.30	4.27	4.25	4.25	4.27

Note: The average oscillator strength of the 1^1B_u state is 0.85 ± 0.01, while the 2^1A_g state is optically dark with an average oscillator strength of $(4.25 \pm 2.50) \times 10^{-5}$.

Table S9: Energy gaps (in eV) at the 2^1A_g-min, between the 2^1A_g state and the 1^1B_u and GS states for all the (non-equivalent) m5 conformers (SA8-CAS(10,10)/RMS-CASPT2).

	**C₂**				**C$_i$**		**C₁**			
	AA	BB	B⁻B⁻	A⁻A⁻	AA⁻	BB⁻	AB⁻	A⁻B⁻	AB	A⁻B
$E_{2^1A_g} - E_{1^1B_u}$	-0.48	-0.75	-0.75	-0.48	-0.49	-0.76	-0.86	-0.85	-0.85	-0.86
$E_{2^1A_g} - E_{GS}$	1.93	1.59	1.59	1.94	1.95	1.59	1.73	1.73	1.73	1.73

Table S10: Relative stability (in kcal/mol) of the m5 conformers at the 2^1A_g-min. (SA8-CAS(10,10)/RMS-CASPT2).

**C$_2$**				**C$_i$**		**C$_1$**			
AA	BB	B$^-$B$^-$	A$^-$A$^-$	AA$^-$	BB$^-$	AB$^-$	A$^-$B$^-$	AB	A$^-$B
0	2.62	2.62	0.02	0.43	2.84	0.92	0.71	0.70	0.92

The following Table S11 reports the electronic configurations(and weights) for the key states responsible for the PA2 band (computed from the 2^1A_g state). Notably, along the relaxation, the PA2 evolves from a two peaks structure to a single, narrower, peak. This is reminiscent of the different PA spectral shapes for p3 (presenting two lobes) and p5 (presenting a single, narrower peak) polyenes PA signal. Not only the spectral shapes, but also the state nature (highlighted by their configurations) and its modification from p3-like at the FC to p5-like at the 2^1A_g-min can be traced from the table.

Table S11: Electronic configurations and weights ($\geq$0.05) of the transitions responsible of the PA2 for the 2^1A_g state.

Structure	Transition(s) [Osc. Str.]	Electr. Config.s (Weight)
2^1A_g-min (p3)	3.40 [0.30]	χ_1 (0.34)<br>χ_2 (0.26)<br>χ_4 (0.26)
	4.10 [0.21]	χ_3 (0.58)<br>χ_1 (0.08)<br>χ_2 (0.08)
2^1A_g-min (p5)	3.32 [0.98]	χ_3 (0.23)<br>χ_1 (0.13)<br>χ_2 (0.13)<br>χ_4 (0.11)
	3.54 [0.06]	χ_3 (0.33)<br>χ_2 (0.20)<br>χ_1 (0.12)
FC	2.68 [0.21]	χ_3 (0.32)
	3.25 [0.24]	χ_3 (0.24)
2^1A_g-(BLA-relaxed)	2.82 [0.37]	χ_2 (0.23)<br>χ_1 (0.11)<br>χ_4 (0.09)
	3.00 [0.27]	χ_3 (0.42)<br>χ_5 (0.14)<br>χ_2 (0.09)
2^1A_g-min	2.77 [0.72]	χ_3 (0.25)<br>χ_2 (0.12)<br>χ_4 (0.12)<br>χ_1 (0.10)
	2.90 [0.03]	χ_3 (0.26)<br>χ_2 (0.26)<br>χ_1 (0.06)<br>χ_5 (0.05)

Definitions:
$\chi_1 = H \rightarrow L + 2$
$\chi_2 = H - 2 \rightarrow L$
$\chi_3 = H - 1 \rightarrow L + 1$
$\chi_4 = H \Longrightarrow L, L + 1$
$\chi_5 = H - 1, H \Longrightarrow L$

Table S12: BLA values of the structures along the scan in the central panel of Figure 4a in main text.

	**P3**	**P5**	**M5**
0%	0.11	0.09	0.09
25%	0.06	0.06	0.07
50%	0.02	0.03	0.04
75%	-0.03	-0.01	0.01
100%	-0.07	-0.04	-0.02
